# Which pathways trigger the role of complement in ischaemia/reperfusion injury?

**DOI:** 10.3389/fimmu.2012.00341

**Published:** 2012-11-19

**Authors:** Conrad A. Farrar, Elham Asgari, Wilhelm J. Schwaeble, Steven H. Sacks

**Affiliations:** ^1^MRC Centre for Transplantation, Division of Transplantation Immunology and Mucosal Biology, King’s College London School of Medicine at Guy’s, King’s College and St Thomas’ HospitalsLondon, UK; ^2^Department of Infection, Immunity, and Inflammation, Leicester UniversityLeicester, UK

**Keywords:** ischemia, reperfusion, MBL, kidney, complement

## Abstract

Investigations into the role of complement in ischemia/reperfusion (I/R) injury have identified common effector mechanisms that depend on the production of C5a and C5b-9 through the cleavage of C3. These studies have also defined an important role for C3 synthesized within ischemic kidney. Less clear however is the mechanism of complement activation that leads to the cleavage of C3 in ischemic tissues and to what extent the potential trigger mechanisms are organ dependent – an important question which informs the development of therapies that are more selective in their ability to limit the injury, yet preserve the other functions of complement where possible. Here we consider recent evidence for each of the three major pathways of complement activation (classical, lectin, and alternative) as mediators of I/R injury, and in particular highlight the role of lectin molecules that increasingly seem to underpin the injury in different organ models and in addition reveal unusual routes of complement activation that contribute to organ damage.

## INTRODUCTION

Complement is a member of the innate immune system and is comprised of both soluble proteins and membrane-bound receptors that are activated following invasion of foreign pathogens. The effector molecules that are generated have diverse biological activities, namely, defense against bacterial infection through opsonization; activation of leukocytes; removal of immune complexes and apoptotic cells (Janeway and Medzhitov, 2002); and the augmentation of B cell and T cell-mediated immunity (Pratt et al., 2002; Lee et al., 2005). However, it has clearly been demonstrated over a number of years that complement activation has a deleterious effect in a number of inflammatory conditions, including the rejection of solid organ transplants – effects that have been described in a number of organs, such as the lung, liver, heart, and kidney (Weisman et al., 1990; Eppinger et al., 1997; Zhou et al., 2000; Lehmann et al., 2001).

There are three different pathways that initiate the complement cascade, namely the classical, alternative, and the lectin pathways. The classical pathway is initiated once antibody-antigen complexes bind the classical pathway recognition subcomponent C1q, which forms the multimolecular C1 complex with the classical pathway-specific serine proteases C1r and C1s. The alternative pathway is activated by distinct carbohydrate or lipid motifs on pathogens or altered self molecules, leading to recruitment of C3 and factor B. Mannan-binding lectin (MBL) is one of five different lectin pathway-specific carbohydrate recognition molecules in man that associate with lectin pathway-specific serine proteases to drive complement activation. MBL shares a high degree of structural homology with C1, the multimolecular complex that initiates the classical activation pathway of complement. MBL binds to carbohydrate residues on microorganisms, or altered-self endogenous ligands that arise in pathological conditions such as ischemia/reperfusion (I/R) injury. MBL forms complexes with MBL-associated serine proteases (MASPs), which share a high degree of structural homology with the classical pathway serine proteases C1r and C1s. Three different forms of MASPs (MASP-1, MASP-2, and MASP-3) have been described, and of those, MASP-1 and MASP-3 are encoded by a single structural gene (Thiel, 2007). When lectin pathway activation complexes bind to microbial carbohydrates or acetylated ligands, MASP-1 and MASP-2 are converted into their enzymatic active form. Of those, only MASP-2 can translate binding into complement activation, by subsequent cleavage of the complement components C4 and C4b-bound C2, to form the lectin pathway C3 and C5 convertase complexes C4b2a and C4b2a(C3b)_n_ respectively. MASP-1 can cleave C4b-bound C2, but not C4, therefore the lectin pathway activation route is deficient in the absence of MASP-2 (Schwaeble et al., 2011). MASP-1 can augment MASP-2 functional activity by cleaving C2 and possibly enhancing complement activation by conversion of MASP-2 into the enzymatic active form, but it cannot compensate for the loss of MASP-2 functional activity. A recent study using MASP-1-specific inhibitory peptides implies an essential role of MASP-1 in aiding the activation of MASP-2 (Heja et al., 2012), a hypothesis that is not supported by the phenotype of serum of MASP-1 and MASP-3-deficient mice, as this serum clearly shows reduced but marked lectin pathway functional activity mediated by residual MASP-2 (Schwaeble et al., 2011). The convergence point of all three pathways is the activation of C3, an abundant plasma component that is converted into C3a and C3b via a C3 convertase, a process that leads to the subsequent formation of membrane attack complexes. Though the liver is the main site of complement C3 synthesis (Alper et al., 1969), a wide range of soluble complement proteins, including C3, are also produced by extra-hepatic synthesis. The synthesis of many soluble complement proteins in a variety of cells and tissues is comprehensively outlined in a recent review (Li et al., 2007). The purpose of this article is to focus on the role of complement in inflamed whole organs, particularly transplanted ischemic kidney, with emphasis on emerging knowledge of the relative contribution of the complement activation pathways to tissue injury.

## RENAL I/R INJURY

Ischemia/reperfusion injury manifests rapidly and is the product of tissue hypoxia and production of free radicals following the introduction of oxygenated blood to an oxygen-deprived kidney (Grace, 1994). The tissue architecture of an ischemic organ has significant bearing on the nature of the inflammatory reaction that mediates damage. In ischemic kidney the primary area of complement-mediated attack appears to be the tubulo-interstitium within the corticomedullary junction (Bonventre, 1993). Here, significant tubulo-interstitial damage via the alternative pathway of complement activation has been demonstrated in both native kidney (Zhou et al., 2000) and human transplanted kidney (Thurman et al., 2005). In both rodent renal allografts and syngeneic grafts, reperfusion injury affects the proximal tubules in the corticomedullary region (Pratt et al., 2000; Farrar et al., 2006).

## LOCAL RENAL COMPLEMENT PROTEIN EXPRESSION

### RENAL SYNTHESIS OF COMPLEMENT PATHWAY COMPONENTS

Following ischemic insult and subsequent reperfusion, renal cells targeted by complement activation are also capable of significant complement protein biosynthesis themselves. Complement C3 and/or C4 can be expressed by proximal tubular epithelial cells (Brooimans et al., 1991), glomerular epithelial cells (Sacks et al., 1993; Zhou et al., 1993), endothelial cells (Sheerin et al., 1997), and glomerular mesangial cells (Sacks et al., 1993). Complement proteins can be produced by the liver in large amounts, which raises a question as to the significance of local renal complement production during renal transplantation. It can be speculated that local production by both resident cells (Sacks et al., 1993) and infiltrating leukocytes (Botto et al., 1992) enhances the speed of reaction leading to an inflammatory response and tissue damage following reperfusion insult. Additionally, tissue-specific regulation of complement proteins at sites of inflammation may confer advantages over peripheral gene expression. This idea is given credence by the observation that interleukin-1 (IL-1) can potently stimulate renal complement production in the absence of any effect on hepatic synthesis (Falus et al., 1987). In fact, most of the complement proteins of both the classical and alternative activation pathways can be produced within the kidney (Passwell et al., 1988; Song et al., 1998). To date, only hepatocytes have been shown to synthesize MBL, except for the rat, in which extra-hepatic expression of MBL protein has been reported within the renal corpuscle and distal tubules of the kidney (Morio et al., 1997). It has also been detected in the small intestine of mice (Wagner et al., 2003), whereas the biosynthesis of MASP-2 strictly occurs in hepatocytes and is undetectable in any extra-hepatic tissue (Stover et al., 2004).

### COMPLEMENT REGULATORY PROTEINS EXPRESSED WITHIN THE KIDNEY

Regulators of complement activation are expressed on most cells and tissues, providing crucial protection from autologous complement activation and deposition. These complement control proteins are expressed in a variety of renal cells (Nangaku, 1998) and include proteins such as CD46 (membrane cofactor protein; MCP) and CD55 (decay accelerating factor; DAF). DAF functions by enhancing the dissociation of Bb/C2a from C3 convertases and MCP acts as a cofactor for factor I-mediated cleavage of C3b and C4b. Both regulatory components target the activity of every C3 and C5 convertase complex containing either C3b or C4b. Under physiological circumstances MCP and DAF prevent excessive activation of complement, and it is of interest to note that the hypoxia-sensitive renal corticomedullary junction displays low expression of DAF (Cosio et al., 1989). This may go some way to explaining why this region of the kidney is particularly susceptible to complement-mediated attack. Rodent complement regulator Crry, a homologue of human CR1 (Quigg et al., 1998) is expressed within the corticomedullary junction. Under inflammatory conditions associated with ischemia and reperfusion in native kidney, Crry expression re-polarizes from the basolateral surface of the renal tubules to within the tubule lumen (Thurman et al., 2006), exposing tubules to complement-mediated attack. An increase in C3 mRNA expression was also observed, suggesting that loss of complement regulation and/or increased local complement production contributes to the pathogenesis of reperfusion injury (Thurman et al., 2006). A more closely defined role for locally-expressed Crry using Crry-deficient kidneys has been identified. When transplanted into Crry-sufficient recipients, deficient kidneys are subject to more severe renal damage, exemplified by unrestricted C3 activation, increased tubular damage and fibrosis, with a significant influx of polymorphonuclear cells (Bao et al., 2007). More recently it has been demonstrated that interaction of complement regulator factor H with the surface of tubular epithelial cells is required to curb complement deposition following renal I/R injury, as mice treated with aninhibitor of factor H displayed severe tubular injury (Renner et al., 2011).

## EFFECTORS OF COMPLEMENT-MEDIATED DAMAGE FOLLOWING RENAL ISCHEMIA/REPERFUSION INSULT

In renal transplantation, organ reperfusion and cell-mediated immune mechanisms directed at the kidney, as well as invasion by bacteria, are associated with worse transplant outcome. These pathological conditions predominantly affect the renal tubules, where there is significant complement deposition. In native kidney, animals with individual deficiency of C3, C5, or C6, demonstrated significant reduction in reperfusion damage, with corresponding reduction in renal failure (Zhou et al., 2000). The most striking protective effect was seen in the absence of C3, and further studies in the absence of C5 or C6 suggested that reperfusion injury is dependent on the formation of the lytic effector molecule MAC (Zhou et al., 2000). Indeed, further evidence supports a role for both effectors C5a and MAC formation in mouserenal reperfusion injury (Arumugam et al., 2003; de Vries et al., 2003).

## LOCALLY PRODUCED C3 IN I/R INJURY

Regarding the contribution of locally produced C3 within the kidney, C3 mRNA is up-regulated within 2h of reperfusing ischemic rat kidney (Takada et al., 1997). Kidney swap experiments between complement C3-sufficient and C3-deficient mice have been informative. It was demonstrated that locally synthesized C3, produced by and deposited on the basolateral surface of proximal tubule cells, was essential for complement-mediated reperfusion injury in transplanted kidney (Farrar et al., 2006). Significant involvement of circulating C3 was not observed, a phenomenon that could indicate poor penetration of hepatic C3 (C3 is a 180-kD protein), and/or an excessive production of complement within the inflamed kidney. Indeed, the period of cold ischemia is closely associated with an increase in renal C3 mRNA (Pratt et al., 2000; Farrar et al., 2006). It is worth noting that the effect of local renal C3 production is still observed in allograft models, but it is difficult to disentangle the effects of C3 on allograft rejection from those associated with I/R injury alone (Pratt et al., 2002).

## WHICH ARE THE MAIN COMPLEMENT ACTIVATION TRIGGER PATHWAYS IN WHOLE ORGAN I/R INJURY?

Since C3 has been shown to be essential for the formation of C5a and C5b-9 in animal models of reperfusion injury, a key question concerns the trigger pathways that lead to the cleavage of C3 at the affected tissue site. Studies in several organ models (of I/R damage) have yielded potentially conflicting results about the contribution of different pathways to the cleavage of C3. Here we review the evidence for involvement of classical, lectin and alternative pathway activity, which highlights an emerging role for lectin pathway activation.

### SKELETAL AND INTESTINAL I/R INJURY

In skeletal muscle and the intestine, I/R injury is associated with marked complement activation and endothelial cell damage within the vasculature and subsequent tissue necrosis (Weiser et al., 1996; Zhao et al., 2002). Limited injury was observed in an intestinal reperfusion injury model suggesting a dependency on C2, MBL and activation of the lectin pathway for injury, with no significant contribution from C1q and classical pathway activation or alternative pathway involvement (Hart et al., 2005). However, findings in a similar animal model of intestinal reperfusion injury suggested that the observed damage was dependent upon IgM-mediated activation of the classical pathway (Williams et al., 1999; Zhang et al., 2004). Recently, a more detailed understanding of the mechanism of lectin pathway activation in intestinal I/R injury has been described (Zhang et al., 2006), in which MBL was shown to bind naturally occurring IgM at the site of injury, leading to lectin pathway activation with no involvement of the classical pathway. Furthermore, in a study of skeletal reperfusion injury, it was initially suggested that inflammation was mediated exclusively through an effect of classical pathway activation (Weiser et al., 1996). However, with the availability of both C1q-deficient mice and MBL-deficient mice, it now seems clear that activation of both lectin and classical pathways is responsible for the range of inflammatory responses observed. Muscle injury is dependent on lectin pathway activation, with remote pulmonary injury and vascular permeability (manifest by edema), being dependent on classical pathway activation (Chan et al., 2006). It has been conclusively demonstrated by surface plasmon resonance that human MBL binds IgM and subsequent treatment of human endothelial cells *in vitro* with IgM, MBL and MASP-2 directly activated and deposited C4 (McMullen et al., 2006). More recently, using the same model of intestinal I/R injury as described earlier (Zhang et al., 2006), in which the mesenteric artery is clamped, tissue injury was more firmly attributed to activation of the lectin pathway, as MBL was present in association with naturally occurring IgM. The injury was not mediated by alternative pathway activation when the same injury protocol was applied to factor B knockout mice (Lee et al., 2010). This naturally-occurring antibody was earlier found to be a self-reactive IgM capable of mediating intestinal reperfusion injury (Zhang et al., 2004). Presence of this IgM has led to the discovery of two distinct self-antigens (ischemia-specific antigens), namely type A and C non-muscle myosin heavy chain (NMHC-II; Zhang et al., 2006) and more recently, actin cytoskeleton has been shown to bind IgM during ischemia, leading to reperfusion injury (Shi et al., 2009). These recent observations are very exciting as they have uncovered a previously unresolved role for IgM in intestinal reperfusion injury, the hallmark of which is characterized by binding of IgM to endogenous ligands exposed upon injury, with direct activation of the lectin pathway without involvement of the classical pathway.

### MYOCARDIAL I/R INJURY

An association of both C3 and MBL deposition in ischemic rodent heart was observed over a decade ago (Collard et al., 2000). Subsequently, it was shown that blockade of rat MBL with a therapeutic antibody reduced the extent of myocardial reperfusion injury (Jordan et al., 2001). Moreover, MBL deficiency conferred protection from injury, whereas classical pathway activation appeared not to be involved in mediating injury, as C1q-deficient mice were not protected (Walsh et al., 2005). This finding echoed observations in the intestine, where presence of C1q-mediated classical pathway activation was not a requirement for injury (Hart et al., 2005). Further insight into the mechanisms causing myocardial reperfusion injury was provided through reconstitution experiments in triple knockout mice, in which injury was dependent upon both naturally occurring IgM and MBL-mediated complement activation (Busche et al., 2009). Of particular interest is the observation that diabetic patients, who are at risk of cardiomyopathy, may benefit from transient blockade of MBL, as MBL-knockout mice are protected from diabetes-induced myocardial reperfusion injury (Busche et al., 2008). Interestingly, as in I/R injury of the intestine, it is emerging that there is significant contribution to pathophysiology through an interaction between naturally-occurring IgM and lectin pathway activation (Busche et al., 2009).

### KIDNEY I/R INJURY

That C3 is a crucial mediator of renal reperfusion injury is not in dispute. This seems unsurprising, given that C3 is the central protein of the complement cascade, the point of convergence for all three recognized activation pathways. Classical pathway activation is currently not regarded as a requirement for renal reperfusion injury, as it has been demonstrated that injury is independent of both IgG and IgM (Park et al., 2002) and deficiency of C4 was non-protective both in native kidney (Zhou et al., 2000) and renal transplant models (Lin et al., 2006) in the mouse. An absence of classical pathway activation in the kidney is perhaps not surprising as it seems unlikely that there would be adequate transfer of IgM from the renal capillaries to the interstitium, the principle target of reperfusion injury. Damage within the kidney is manifest by significant MAC deposition that was curtailed in the absence of C3 (Zhou et al., 2000). This MAC deposition hinted at the involvement of alternative pathway activation as a mediator of renal reperfusion injury. In addition, no protective effect was found in the complete absence of C4, suggesting that the phenotypic injury did not result from classical or lectin pathway activation, since C4 is common to both (Zhou et al., 2000). However, the recent discovery that the lectin pathway shows residual functional activity in C4-deficient mouse and human sera may explain the persistence of complement-mediated I/R injury in C4-deficient subjects (Schwaeble et al., 2011). A role for contribution of the alternative activation pathway was demonstrated in factor B knockout mice, which were afforded significant protection from reperfusion injury when compared to mice replete with factor B (Thurman et al., 2003). Lectin pathway involvement in renal reperfusion injury was suggested following a study in which both mouse kidney (damaged by I/R injury) and human renal transplant biopsies displayed significant deposition of MBL-A and MBL-C, components that were found to co-localize with complement C3 (de Vries et al., 2004). Using transgenic mice deficient in both MBL-A and MBL-C, contribution of lectin pathway activation to renal I/R injury was demonstrated (Moller-Kristensen et al., 2005). The observed protective phenotype was readily reversed following reconstitution of the mutant mice with recombinant MBL (Moller-Kristensen et al., 2005). In a pig model, inhibition of classical and lectin activation pathways following the administration of recombinant human C1-inhibitor (rC1INH), conferred protection from renal reperfusion injury (Castellano et al., 2010). Components of the lectin pathway (MBL and MASP-2) were abundantly deposited in the damaged kidney, with sparse C1q deposition, suggesting a predominance of lectin pathway activity (Castellano et al., 2010). More recently, an entirely new concept of lectin pathway participation in renal reperfusion injury suggested that MBL is internalized by damaged tubules which are then subject to apoptosis through an MBL-mediated mechanism. This injury was foundto be independent of an effect of complement (van der Pol et al., 2012).

### UNORTHODOX ROUTES OF COMPLEMENT ACTIVATION VIA LECTIN PATHWAY IN I/R INJURY

As most recently described (Turner, 2003), there has been significant interest in the contribution of the lectin pathway to I/R injury. In models of intestinal, skeletal and renal I/R injury, significant contribution of the lectin activation pathway was observed. It is of particular interest that the lectin pathway may trigger inflammatory responses within the pathophysiology of I/R injury by unconventional and as yet undefined bypass activation events. This may have an impact on our current understanding of the mechanism of lectin pathway activation in reperfusion injury. Although C4 is traditionally considered a requirement for lectin pathway-mediated cleavage of C3 (Thiel et al., 1997; Vorup-Jensen et al., 2000), there is clear evidence for the existence of so called “bypass” pathway activation events, leading to residual activation in the absence of certain cascade components caused by inherited or gene-targeted deficiencies (Degn et al., 2007). The existence of a C4-bypass pathway was first reported in C4-deficient guinea pig serum (May and Frank, 1973). Recent work has provided remarkable insight into the role of lectin pathway components in models of both cardiac and intestinal I/R injury in mice (Schwaeble et al., 2011). In both pathological states, injury was induced via MASP-2-mediated lectin pathway activation. The report also demonstrated that C4 was not required, suggesting the presence of a previously unrecognized C4-independent MASP-2-dependent bypass route of lectin pathway cleavage of C3. These findings may explain the lack of protection of C4 deficiency in both native renal ischemia and the model of renal allograft rejection discussed earlier (Zhou et al., 2000; Lin et al., 2006). This C4-independent MASP-2-dependent bypass mechanism has recently has been confirmed in a renal transplant model (Farrar et al., 2009). Importantly, these recent findings in different organ models of I/R injury highlight MASP-2 as an early trigger point in a specific complement activation pathway, which may prove to be an attractive therapeutic target in ischemic inflammatory settings. As to the role of MASP-1 or MASP-3 in complement-mediated I/R injury, gene-targeted mice deficient of both MASP-1 and MASP-3 are not protected from injury, indicating that neither MASP-1, nor MASP-3 contribute to MASP-2-dependent reperfusion injury (W. Schwaeble, unpublished data).

## MBL AND LECTIN PATHWAY OF COMPLEMENT ACTIVATION IN HUMANS

A link between deficiency of MBL and susceptibility to infection in both man and rodents has been established (Eisen and Minchinton, 2003; Shi et al., 2004). In experimental models of renal reperfusion injury, MBL deficiency in rodents (Moller-Kristensen et al., 2005) and lectin pathway inhibition in pigs (Castellano et al., 2010) led to improved renal function. In human transplant recipients suffering from delayed graft function (DGF) after transplantation, there was a positive association with MBL pathway products (de Vries et al., 2004). Moreover, in human kidney transplantation, genetically-determined low levels of pre-transplant serum MBL correlated with significantly improved transplant outcome (Berger et al., 2005). A similar study looking at simultaneous pancreas and renal transplantation again found that low levels of circulating MBL correlated with improved long-term kidney survival (Berger et al., 2007). However, a recent study of a large cohort of donor and recipient MBL and MASP-2 genotypes failed to confirm a link between genotype and allograft function following kidney transplantation (Damman et al., 2012). Hence to date, from a limited number of studies in humans, there is no conclusive agreement upon the importance of genetic variation of lectin pathway components in ischemic renal allografts, although this remains a subject of much interest as a possible means to stratify patient groups according to expected outcome of the transplant.

## CONCLUDING REMARKS

This brief overview of the role of complement activation in the pathogenesis of whole organ ischemia highlights a previously unrecognized predominant role of lectin molecules in several organ models of I/R injury. One possible trigger mechanism leading to activation of the lectin pathway is the binding of natural IgM to epitopes exposed on ischemic tissue, with no involvement of previously implicated classical pathway activation. The alternative pathway is implicated in some instances, for example following renal ischemia, and may serve to amplify the cleavage of C3 and subsequent evolution of injury, following initiation through the lectin pathway, or could be seen as an independent event. This prevalent role of lectin pathway activation makes it an attractive target for therapeutic intervention, with MASP-2 a potential candidate because of an absolute requirement of MASP-2 for lectin pathway activation in the pathogenesis of renal reperfusion injury. Although there are some conflicting results arising from early studies of the contribution of lectin pathway variants in the clinical setting, these findings do not preclude the investigation of therapeutic blockade of lectin pathway activation during the immediate post-transplant stage, where ischemic events have a significant impact upon renal allograft function.

## Conflict of Interest Statement

The authors declare that the research was conducted in the absence of any commercial or financial relationships that could be construed as a potential conflict of interest.
